# Is There An “Ideal Instagram Face” for Caucasian Female Influencers? A Cross-Sectional Observational Study of Facial Proportions in 100 Top Beauty Influencers

**DOI:** 10.1093/asjof/ojae085

**Published:** 2024-10-10

**Authors:** Rafael Loucas, Bruno Sauter, Marios Loucas, Sebastian Leitsch, Omar Haroon, Aljosa Macek, Silke Graul, Alexander Kobler, Thomas Holzbach

## Abstract

**Background:**

In the digital arena, wherein younger generations predominantly spend their time, social media continues to determine what is considered beautiful. Social media, particularly Instagram (Meta, Menlo Park, CA), is becoming a prominent aspect of the plastic surgeon–patient relationship. Therefore, the beauty ideal escalates without any barriers and breaks. The majority of influencers look alike. This sets a new trend for a beauty ideal.

**Objectives:**

In this study, the authors aimed to analyze the facial proportions of 100 top female beauty influencers, to evaluate them for identifying the “ideal Instagram face,” and to determine whether there existed a deviation from the standard golden ratio.

**Methods:**

The authors identified the top 100 beauty influencers, according to the latest rankings of November 2022. A detailed facial analysis has been conducted using the Fiji biomedical image analysis software. The primary outcome parameters included facial proportions such as lip ratio, Ricketts’ line, and nasal dimensions. Secondary outcomes comprised BMI, age, and ethnicity. The results were collected and analyzed descriptively using graphs and statistics.

**Results:**

Complete datasets were obtained from the top 100 female beauty influencers with a mean age of 31.3 ± 6.3 years (range, 24-38 years). The majority of influencers were Americans, followed by Germans and British. Based upon the mean of the aforementioned parameters, we could design the ideal Instagram face. There was no significant difference between the golden ratio and the new trend of the ideal Instagram face.

**Conclusions:**

Despite the existence of varied ethnic population groups and nonstandard measurements, the ideal Instagram face represents today's ideal trending face. The ideal Instagram face is symmetrical, matching the golden ratios, with a small and neat nose, full and lush lips, high cheekbones, as also a sharp and chiseled jawline. Further studies on this topic, involving a greater number of influencers with standardized measurements, should be advocated to identify the ideal facial proportions. This will lead to improvements in invasive and noninvasive cosmetic treatments.

**Level of Evidence: 5 (Diagnostic):**

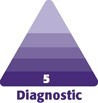

In the digital arena, wherein younger generations predominantly spend their time, social media persistently shapes societal perceptions of beauty.^1-6^ The proliferation of image-centric social media platforms has catalyzed a paradigm shift in beauty standards, transcending traditional media influence.^6,7^ Social media, particularly Instagram (Meta, Menlo Park, CA), is becoming a prominent part of the plastic surgeon–patient relationship.^1,2,8^ This evolution has profound implications for self-perception and aesthetic ideals, prompting a more nuanced understanding of the subjective and evolving nature of beauty standards. Around 95 million photographs and videos are uploaded to Instagram daily.^2,3,9^ These uploads, increasingly manipulated through digital tools, present an altered reality that shapes public perceptions of beauty.

Since the launch of Instagram, just over a decade ago, an entire industry has grown out of the desire to present oneself in the best possible light.^2,3,9-11^ Filters and editing tools that give the appearance of a mirror-smooth skin or enlarge the eyes and diminish nose-size assist in offering a better image.^12,13^ The widespread use of such tools suggests a growing divergence between natural and digitally enhanced aesthetics. The Facetune (Lightricks Ltd, Tel Aviv, Israel) program, which can be used to slim faces or perfect jawline contours, has sold millions of copies. This underscores a significant shift toward digital aesthetic enhancement in everyday social media interactions. Almost everyone who makes their living looking good on social media or anywhere else uses such enhancement techniques.^10,12,13^ Such trends raise questions about the impact of digital beauty standards on societal perceptions of attractiveness. Additionally, most influencers look alike and propagate a particular ideal of beauty. Thus, digital facial optimization has been changing rapidly over the last couple of years, thereby establishing a new trend for a beauty ideal.^6,12,14,15^ Building on established psychological theories, Jones et al describe “Cognitive Averaging” as a process wherein exposure to diverse facial types influences perceptions of beauty, leading to an averaged concept of the “ideal.”^6,12,14,15^ This evolutionary psychology concept highlights the human tendency to prefer phenotypic normalcy, considered an indicator of genetic health. Similarly, the principle of Koinophila, which denotes an attraction to average features, is believed to signal a robust genetic background, contributing to the organism's overall fitness.^6,15^

Walker et al^1^ attempted to explore whether exposure to images with facial cosmetic enhancements increased the desire for cosmetic surgery. They reported that viewing images of women who had undergone cosmetic enhancements affected young women’s desire for cosmetic surgery, especially if they spent a significant amount of time on social media, followed several accounts, and were less satisfied with their appearance.^1^

Physical appearance is an important aspect of personal identity, and its relation to individuals’ self-perception begins early in life.^15,16^ Although perceptions of attractiveness are guided by innate preferences such as symmetry, social media can also influence perceptions of beauty.^14,15,17,18^ Thus, we observe that the ideal of beauty is changing faster than ever before, especially through social media, and in particular, through the Instagram influence. The latter revolves chiefly around images; it makes far lesser use of written text, in comparison with other social networking sites, such as Facebook, Tiktok, and Twitter.

In light of evolving digital trends, we hypothesize that the “ideal Instagram face” symbolizes contemporary beauty standards, shaped significantly by the visibility of beauty influencers on platforms such as Instagram. This hypothesis is underpinned by empirical observations from recent research which suggest that social media not only reflects but also establishes beauty trends.^4^

Studies have found that social media use can impact the desire for cosmetic surgery and treatments.^5,6,12,19,20^ Moreover, the demand for facial aesthetic treatments has increased over the last 20 years.^21-23^ With this increase, it is becoming more important for plastic surgeons to be aware of the current beauty trends and ideal facial proportions.^22,23^ Recent research underscores the transformative influence of social media on aesthetic standards, particularly the emergent phenomenon known as the “Instagram face.”^4-7^ This face, often characterized by specific facial features and proportions, reflects a convergence of digital and traditional beauty norms. Our study has situated itself within this evolving landscape, seeking to empirically investigate and quantify these changes. Further, the advent of sophisticated digital-editing tools and filters has amplified the impact of social media on beauty ideals, blurring the lines between virtual and physical aesthetics.

Measurements of facial proportions were introduced by the Greeks in the Classical Canons, and later adopted by Renaissance artists.^24^ During the European Renaissance, renowned artists and architects used an equation known as the “golden ratio,” to map out their masterpieces.^24,25^

Thousands of years later, scientists adopted this mathematical formula to help explain why some people were considered beautiful.^25^ This ratio is 1:1.618.^24,25^

In the present study, the authors have attempted to analyze the facial proportions of 100 top female beauty influencers, to evaluate them for identifying and designing the ideal Instagram face, and to determine whether there existed a deviation from the standard golden ratio.

## METHODS

### Study Design and Study Population

In this retrospective observational cohort study, the authors analyzed the facial proportions of 100 Instagram influencers. The top 100 beauty influencers were selected according to their rankings in the list of top Instagram beauty influencers.

Based upon their social media accounts and the numbers of their followers, these influencers were publicly listed as the “top beauty/fashion Instagram influencers on Instagram,” in November 2022.

For the selection of our sample population, we referred to the rankings on HypeAuditor (Indianapolis, IN; https://hypeauditor.com/top-instagram-beauty/) as on November 2022 to identify the top 100 beauty influencers. This source provided a reliable method for determining prominent individuals within the beauty sector on Instagram based on metrics such as follower count, engagement rate, and content quality. Given the public nature of the content shared by these influencers, our study’s use of their publicly available images for analysis adheres to fair use guidelines. The influencers’ status as public figures legally allows for the analysis of their public digital presence without infringing personal privacy rights.

### Participant Selection

Our database included data about the “100 top beauty Instagram influencers.” The following data were collected additionally: ethnicity, gender, age, and number of followers on Instagram. The Instagram influencers were selected from the ranking list of top beauty Instagram influencers of November 2022. To be included in the study, Instagram influencers were required to have a minimum of 250,000 followers, belong to the category of beauty/fashion influencers, and have front and side profile pictures of their faces. We excluded Instagram influencers of Asian and African ethnicities, transgender persons, males, and Instagram influencers listed under other categories.

### Two-Dimensional Assessment of Facial Proportions

A total of 100 front and side profile pictures were identified on Instagram. The individual images were saved and cropped through macOS Ventura 13.3.1 and Safari 16.4.1 (Apple Inc., Cupertino, CA). Images were selected from each influencer's profile based on high resolution, clear facial visibility, and natural lighting, ensuring consistency across the dataset.

Face analysis was performed using a 2-dimensional (2D) digital measurement assessment software: Fiji by ImageJ2 (Madison, WI), an open-source platform for biological image analysis (version: 2.9.0/1.53t; open-source image processing software).

The measurements were executed in a standardized manner: point-to-point vectors were applied using Fiji software. Fiji software includes an “arrow tool” to measure straight, point-to-point distances over quadratic pixels. By setting a reference distance (the individual's face length from hairline to chin) within each picture to 100%, it was possible to create referring distances (measured vectors) for each individual image and distance (vector) within the same image. To ensure consistency in our measurements, we standardized each image by using the face length from hairline to chin as a reference parameter. This reference length was set at 100%, with all other facial measurements expressed as percentages relative to this baseline.

**Figure 1.**
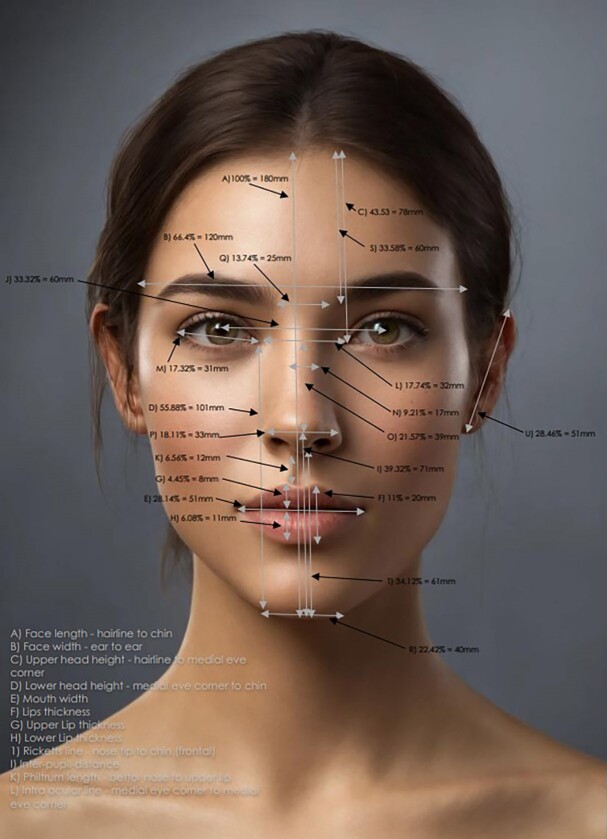
Front profile of the “ideal Instagram face.” Image created using DALL-E, an AI-powered text-to-image model developed by OpenAI (San Francisco, CA).

**Figure 2.**
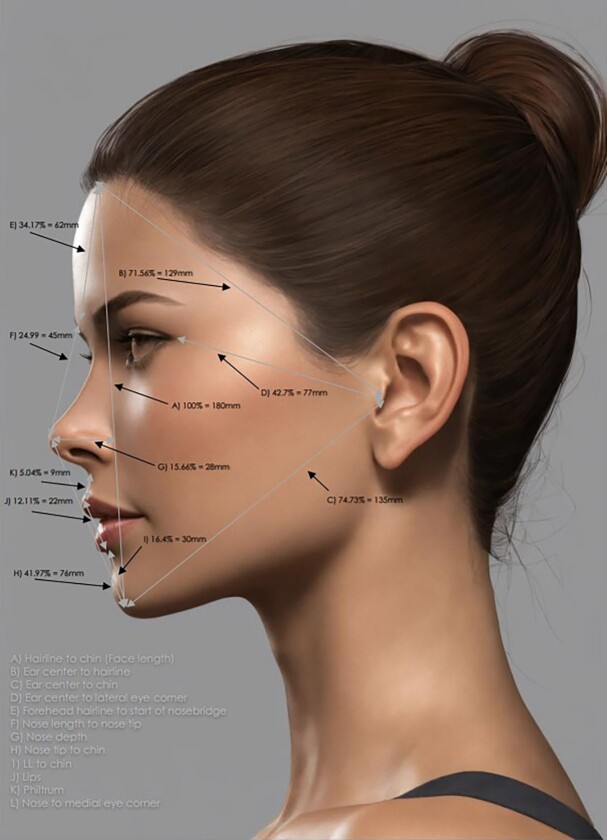
Side profile of the “ideal Instagram face.” Image created using DALL-E, an AI-powered text-to-image model developed by OpenAI (San Francisco, CA).

Vectors measured were as follows: face width, intercanthal distance, the distance between brows, nasal width, eye width, lip width, chin width, face length, forehead length, lower face length, mid-face length, ear length, nose length, philtrum length, lip thickness, upper lip thickness, lower lip thickness, distance from hairline to ear center, distance from ear center to the chin, distance from ear center to lateral canthus, nose depth, length of Ricketts’ line, and the length of the philtrum. These comprehensive measurements are visually represented in Figures 1 and 2. By using a photograph of an influencer, advanced AI software was employed to create a modified image incorporating these measurements, providing a clearer understanding and visualization of the facial vectors mentioned.

### Data Collection, Statistical Analyses, and Literature Review

The Instagram influencers’ data were anonymously collected through the ELO Software/Electronic Data Capture System (ELO Digital Office GmbH, Stuttgart, Germany).

All statistical analyses were performed using SPSS software, version 28.0 (IBM, Armonk, NY). The normal distribution of variables was tested using the Shapiro–Wilk test, and golden ratio proportions were compared through the paired *t*-test (parametric data) or the Wilcoxon rank-sum test (nonparametric distribution). A *P* value of <.05 was considered significant.

## RESULTS

### Participants and Demographics

One hundred female Instagram influencers were selected from the top influencers’ rankings for facial analysis. The mean age of the participants was 31.3 ± 6.3 years (range, 24-38 years). The majority of influencers were Americans, followed by Germans and British (Table 1). All influencers fell under the category of top beauty and fashion influencers. The total number of their followers stood at 13.6 million.

**Table 1.** Facial Proportions Outcomes

Facial proportions (mean)	Measurement (mm)	Reference (mm)
Face width	120 ± 52	136.2 (106-158)^26^ 140 ± 0^27^
Intercanthal distance	32 ± 42	32.0 ± 2.9^28^ 36.36 ± 2.41^29^
Distance between brows	25 ± 24	23.9 ± 3.52^30^
Eye width	31 ± 27	24.27 ± 3.38^30^
Lip width	51 ± 41	49 ± 32^28^
Chin width	40 ± 2.9	—
Face length	180 ± 17	189 (160-220)^26^ 141.72 ± 18.82^30^
Forehead length (trichion–glabella)	60 ± 2.79	64.5 ± 6.9^30,31^ 47.476 ± 6.906^30^
Lower face length (subnasale–menton)	61 ± 62	70 ± 0^26^ 49.60 ± 46.59^30^
Mid-face length (glabella–subnasale)	39 ± 2.71	44.65 ± 6.77^30^
Ear length	51 ± 4.2	60.4 ± 3.2^32^
Nose length	45 ± 4.6	58.39 ± 3.2^29^
Philtrum length	9 ± 1.4	16.2 ± 2.2^28^
Lip thickness	20 ± 3.2	14.8 ± 2.4^28^
Upper lip thickness	8 ± 1.3	8.0 ± 1.1^28^
Lower lip thickness	12 ± 1.32	9.3 ± 1.8^28^
Distance from hairline to ear center	129 ± 14	—
Distance from ear center to chin	135 ± 12	—
Distance from ear center to lateral canthus	13 ± 2	—
Nose depth (nose-tip projection)	28 ± 4.2	36.47 ± 2.11^29^
Distance of Ricketts’ line	76 ± 3.7	—
Eye fissure width	31 ± 3.3	36.80 ± 2.40^29^
Nasal width (nose bridge width)	17 ± 3.1	16.88 ± 1.02^29^
Nose width (nose-ala to nose-ala)	33 ± 3.3	41.66 ± 2.59^29^
Nasal length (nose back length)	39 ± 4.1	32.6 ± 2.6^31^

### Facial Proportions Outcomes

**Figure 3.**
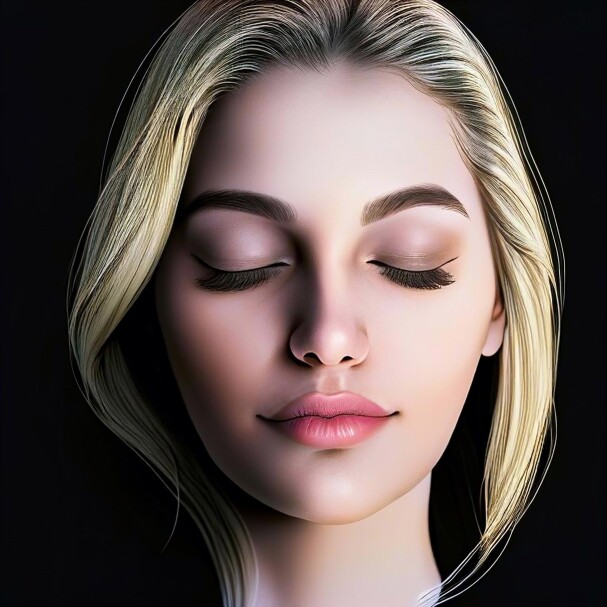
Measurements of front profile. Image created using DALL-E, an AI-powered text-to-image model developed by OpenAI (San Francisco, CA).

**Figure 4.**
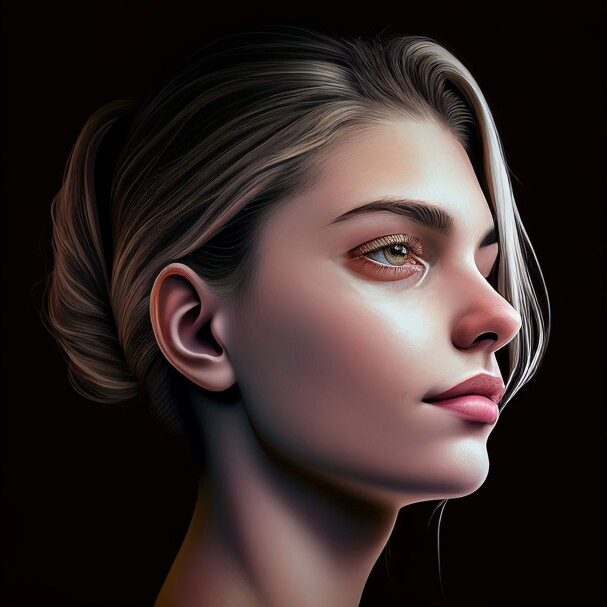
Measurements of side profile. Image created using DALL-E, an AI-powered text-to-image model developed by OpenAI (San Francisco, CA).

**Figure 5.**
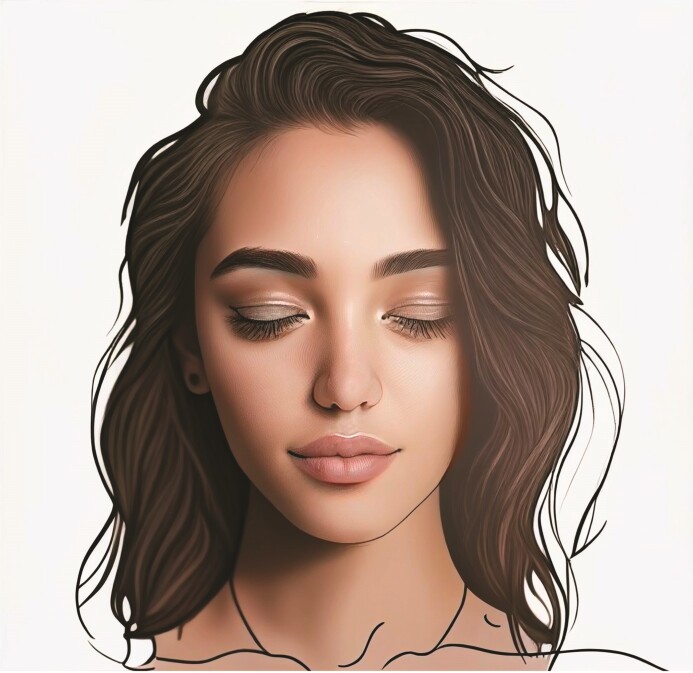
Front profile of the “ideal Instagram face.” Image created using DALL-E, an AI-powered text-to-image model developed by OpenAI (San Francisco, CA).

**Figure 6.**
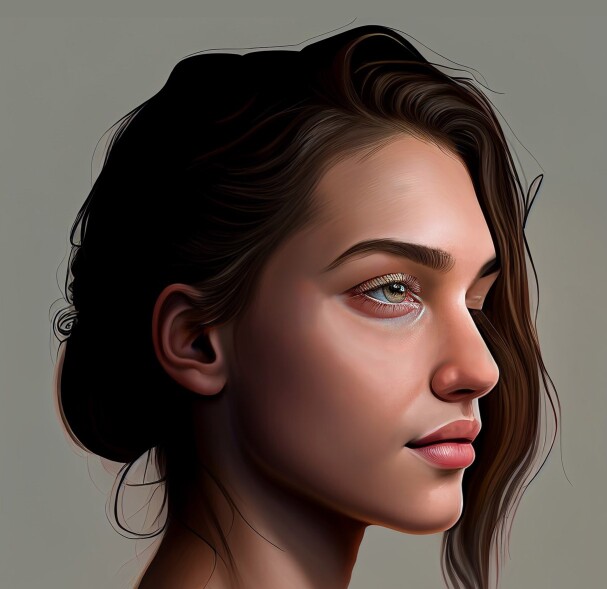
Side profile of the “ideal Instagram face.” Image created using DALL-E, an AI-powered text-to-image model developed by OpenAI (San Francisco, CA).

Using the mean of the above-mentioned facial proportions (Table 1) and DALL-E, a text-to-image AI model created by OpenAI (San Francisco, CA), we were able to generate the following examples of what constitutes an “ideal female Instagram face” (Figures 3-6).

The mean face width was 120 ± 5 mm, mean intercanthal distance was 32 ± 4 mm, mean distance between brows was 25 ± 2 mm, mean nasal width was 33 ± 2 mm, mean eye width was 31 ± 2 mm, mean lip width was 51 ± 4 mm, mean chin width was 40 ± 2.93 mm, mean face length was 180 ± 17 mm, mean forehead length was 60 ± 2.79 mm, mean lower face length was 61 ± 6 mm, mean mid-face length was 39 ± 2.71 mm, mean ear length was 51 ± 4 mm, mean nose length was 45 ± 4 mm, mean philtrum length was 9 ± 1 mm, mean lip thickness was 20 ± 3 mm, mean upper lip thickness was 8 ± 1 mm, mean lower lip thickness was 11 ± 1.32 mm, mean distance from hairline to ear center was 129 ± 14 mm, mean distance from ear center to chin was 135 ± 12 mm, mean distance from ear center to lateral canthus was 13 ± 2 mm, mean nose depth was 28 ± 4 mm, and mean distance of Ricketts’ line was 71 ± 3.58 mm. Additionally, mean eye fissure width was 31 ± 3 mm, mean nasal width (nose bridge width) was 17 ± 3 mm, mean nose width (nose-ala to nose-ala) was 33 ± 3 mm, and mean nasal length (nose back length) was 39 ± 4 mm (Figures 1, 2).

No significant differences were found between the golden ratio and the above measurements (*P* = .68).

## DISCUSSION

Because of the rapid escalation in social media engagement, particularly on platforms such as Instagram, opportunities to engage with social norms influencing the ideal of beauty have been increasingly encouraged.^4-8,12,15^ This trend has significant implications for the field of aesthetic medicine, as it shapes patients’ expectations and preferences.^33-36^

In the “digital arena,” we are constantly exposed to various impulses that shape our perceptions of beauty ideals and unconsciously determine what we perceive as beautiful.^4,5,7-9^ The intersection of technology, social media, and beauty standards presents a complex landscape for both individuals and professionals in the field of aesthetic enhancement.^33,35,36^ Hence, it is fundamentally important to grasp these emerging beauty ideals, so as to support prospective patients with a desire for both optimal and individual aesthetic enhancement. The dynamic nature of these standards necessitates ongoing research and adaptation within the aesthetic medical community. Thus, based on the supporting evidence that social media use can impact the desire for cosmetic surgery and treatments,^13,19,20,37^ the purpose of this study is justified.

This investigation is truly innovative and intriguing, because it addresses how the idea of beauty is changing in the age of digital media, particularly in relation to Instagram influencers.^6,7,13^ It offers a comprehensive understanding of the emerging trends concerning the “ideal Instagram face,” by analyzing the facial proportions of the top 100 female beauty influencers.

The data from our results were analyzed using a 2D digital measurement assessment software, and compared with the previous gold standard for facial aesthetics.^25,38^ Further, assisted by an open-source image processing software, we could design the ideal female Instagram face.

This study is unique by virtue of its assessment of the symmetrical similarities and key common facial features of influencers, and how this new trend aligns with established beauty norms.^22,23,25,38^

Another essential aspect of the study involves its comparison with the standard golden ratio.^23,25,38,39^ No statistically significant differences were found between the conventional gold standard and the newly identified ideal Instagram face (*P* = .68). Although no statistical significance has been claimed, this finding carries significance. It might imply that the new ideals of beauty, propagated by Instagram influencers, are not extremely divergent from the former aesthetic proportions.^22,23,25,38,39^ Therefore, it is evident that, although trends have shifted, the current notions of beauty do not deviate far from the former standards.^22,23,25,38,39^ A detailed comparison of specific facial structures with the golden ratio was provided in the results. Although certain aspects of the ideal Instagram face closely align with this classical proportion, variability was observed across different facial dimensions. This underscores the golden ratio’s relevance as a reference point rather than an absolute standard within the context of contemporary beauty ideals shaped by digital media.

This study also opens avenues for examining the psychological impact of the aforementioned evolving beauty standards.^4-8,13^ It prompts a critical analysis of how these digital trends influence individual self-esteem and body image perception. Additionally, the homogeneity of beauty standards raises concerns about the cultural and ethnic inclusivity in online spaces, highlighting the need for broader representation in the digital beauty narrative.

It should be noted that because Asian and African ethnic groups differ in their facial proportions from Caucasian people, they were excluded from the study.^40-42^ The aim was to achieve homogeneity in the population of Instagram influencers and to focus on the specific aesthetic ideals being promoted by the biggest and most famous influencers. However, this also raises the question whether racial and cultural differences can influence beauty standards, and how radically such cultural diversity and ethnic differences can affect the perceptions of beauty ideals.^40-42^ Future research can examine how varied populations are impacted by these developments and shed light upon them in a broader context.

The quintessential significance of this study is that it sheds light on the ever-evolving landscape of beauty standards from the perspective of the so-called “social arena,” especially Instagram. By precisely analyzing the facial proportions and pinpointing the facial features associated with the ideal Instagram face vis-a-vis the top 100 female beauty influencers, this study offers insights into how plastic-surgical treatments correspond to current aesthetic trends and psychological aspects. Thus, the study demonstrates how visual culture influences contemporary concepts of beauty.^4-7,13^ By understanding the evolving perceptions concerning beauty ideals, service providers will be able to achieve optimal aesthetic results with patient-centered treatment. Further, the results provide a springboard for future interdisciplinary discussions involving disciplines such as psychology, sociology, media studies, and even ethics. Besides, the research findings can be utilized for marketing and advertising purposes and thus be applied to digital design. Aesthetic plastic physicians and marketers can provide content that corresponds to the ideals of beauty, according to the 100 investigated Instagram influencers.^33,35,36^ This will improve the accessibility to the target groups, thereby leading to a better physician–patient relationship and satisfaction, with regard to improved invasive and noninvasive cosmetic procedures.^33,35,36^ Additionally, these findings provide important insights for surgeons seeking to align their practices with contemporary aesthetic trends. By understanding the specific facial proportions that characterize the ideal Instagram face, surgeons can refine their techniques to better match patient expectations. This alignment with current beauty standards can lead to improved patient satisfaction and outcomes, as procedures can be more precisely tailored to the aesthetic preferences popularized on social media. Despite the presented strengths of this study, there are various limitations that one should be aware of. First, as mentioned earlier, it is important to highlight that the influencers were only included based on their audience reach, without considering any ethnic or intercultural deviations. Even though the most prominent 100 influencers may have been included, this does not mean that the full spectrum of beauty trends has been represented. This “sample bias” can cause certain ideals within the population to be overrepresented, thereby altering the diversity of beauty trends that exist on the platform.

Second, the investigation focused on facial proportions but neglected the fact that the included influencers were considered successful, not only because of their physical appearance, but also because of other factors such as charisma, humor, inspiration, and other personality traits. The potential influence of rather simple variables such as hairstyle and make-up or even body proportions has been underrepresented, although understandably difficult to assess. The population studied had an average age of 31.3 ± 6.3 years (range, 24-38 years). However, it was not considered that individuals perceive beauty differently at various stages of life and that this factor may also alter their perceptions. Another weakness of this study is that the currently existing beauty ideals offer only a snapshot of the rapidly evolving digital society. Social media research should be performed on a continuous basis, to capture future changes in the face of constantly changing trends. Furthermore, we recognize the limitations associated with applying the golden ratio as a universal standard of beauty, particularly concerning its relevance across different ethnicities. The golden ratio, while historically significant in aesthetic theory, does not uniformly apply to all populations. To address this, we have included a discussion on the variability in its application, emphasizing the importance of caution when generalizing its use. This consideration is crucial for a more accurate and inclusive understanding of beauty standards, acknowledging the diverse facial features that exist across different ethnic and cultural groups. It is crucial to acknowledge that the ideal Instagram face identified in this study is representative only of the top beauty influencers analyzed and does not reflect global beauty standards. This ideal is specific to a particular subset of online influencers and should not be generalized to the broader population, which encompasses diverse cultural and aesthetic preferences. The study’s findings highlight a trend within a specific online community rather than a universal standard, underscoring the importance of considering a wider range of influences when examining global beauty ideals.

Acknowledging the limitations inherent in using social media photographs for photometric studies is important. These images often vary in lighting, pose, and quality, which can affect the reliability of photometric analyses. Despite these challenges, our study utilized advanced digital measurement techniques to standardize and normalize the data against reference points, improving the reliability and comparability of our findings. This approach underscores the need for ongoing methodological refinement and interdisciplinary research to accurately assess beauty standards in the era of social media. Finally, the study assumed that the preferences and perceptions of their followers were reflected in the beauty ideals promoted by influencers. However, it may be that the followers interacted with influencers for fundamentally different motivations, other than aesthetic reasons.

## CONCLUSIONS

Despite varied ethnic population groups and nonstandard measurements, the ideal Instagram face represents today's ideal trending face. The ideal Instagram face seems symmetrical, matching the golden ratios, with a small and neat nose, full and lush lips, high cheekbones, as also a sharp and chiseled jawline. Further studies on this topic, involving more influencers with standardized measurements, should be advocated to identify the ideal facial proportions, so as to improve cosmetic treatments.
